# Tay-Sachs Carrier Screening by Enzyme and Molecular Analyses in the New York City Minority Population

**DOI:** 10.1089/gtmb.2015.0302

**Published:** 2016-09-01

**Authors:** Nikita Mehta, Gabriel A. Lazarin, Erica Spiegel, Kathleen Berentsen, Kelly Brennan, Jessica Giordano, Imran S. Haque, Ronald Wapner

**Affiliations:** ^1^ Counsyl, South San Francisco, California.; ^2^ Division of Maternal Fetal Medicine, Department of Obstetrics and Gynecology, Columbia University Medical Center, New York, New York.; ^3^ Department of OBGYN-MFM, Columbia Doctors Midtown, New York, New York.

## Abstract

*Background and Aims:* Carrier screening for Tay-Sachs disease is performed by sequence analysis of the *HEXA* gene and/or hexosaminidase A enzymatic activity testing. Enzymatic analysis (EA) has been suggested as the optimal carrier screening method, especially in non-Ashkenazi Jewish (non-AJ) individuals, but its utilization and efficacy have not been fully evaluated in the general population. This study assesses the reliability of EA in comparison with *HEXA* sequence analysis in non-AJ populations. *Methods:* Five hundred eight Hispanic and African American patients (516 samples) had EA of their leukocytes performed and 12 of these patients who tested positive by EA (“carriers”) had subsequent *HEXA* gene sequencing performed. *Results:* Of the 508 patients, 25 (4.9%) were EA positive and 40 (7.9%) were inconclusive. Of the 12 patients who were sequenced, 11 did not carry a pathogenic variant and one carried a likely deleterious mutation (NM_000520.4(HEXA):c.1510C>T). *Conclusions:* High inconclusive rates and poor correlation between positive/inconclusive enzyme results and identification of pathogenic mutations suggest that ethnic-specific recalibration of reference ranges for EA may be necessary. Alternatively, *HEXA* gene sequencing could be performed.

## Introduction

Tay-Sachs disease (TSD; MIM 272800), associated with mutations in *HEXA*, is a neurodegenerative disease caused by accumulation of GM2-ganglioside. TSD most often presents in infancy with regression of skills, intellectual disability, paralysis, dementia, blindness, and death by 5 years of age. Individuals of Ashkenazi Jewish (AJ) ancestry have the highest TSD risk, due to a one in 31 carrier frequency (Gross *et al.*, 2008). Carrier screening originated as an assay measuring hexosaminidase A (Hex A) activity, because below-normal levels indicate carrier status. Molecular analysis later became widely available and historically focused on a defined set of highly prevalent mutations derived from the AJ population. Targeted mutation analysis is less effective for carrier detection in non-AJ populations (Kaback *et al.*, 1993), although TSD does occur in other populations, including the French Canadian (Martin *et al.*, 2007), Cajun (McDowell *et al.*, 1992), Irish (Branda *et al.*, 2004), and Italian (Montalvo *et al.*, 2005) populations.

The American Congress of Obstetricians and Gynecologists (ACOG) presently recommends offering TSD carrier screening to individuals of Ashkenazi Jewish, French Canadian, or Cajun descents (ACOG, 2005), including couples where only one individual reports a high-risk background. However, due to increases in interethnic marriage and multiethnic children rates (Johnson and Kreider, 2010; Jones and Bullock, 2010), identifying at-risk individuals is becoming more difficult. In our laboratory experience, more than 40% of carriers of diseases/mutations most prevalent in the Ashkenazi Jewish population, including those for TSD alone, did not report Jewish ancestry (Lazarin *et al.*, 2013). This demonstrates the limitations of self-reported ethnicity and the potential occurrence of genetic diseases in ethnic groups other than those at “high risk.”

Because targeted mutation analysis for TSD focuses primarily on mutations prevalent in the AJ population and, consequently, has a limited detection rate in the general population (Kaback *et al.*, 1993; Park *et al.*, 2010), enzymatic assays are thought to be more reliable for carrier detection in non-AJ populations (Schneider *et al.*, 2009; Ferreira *et al.*, 2014; Edwards *et al.*, 2015). However, recent data suggest that performance by enzymatic assays may not be optimal in the general/non-AJ population (Strom *et al.*, 2013). Detection rate by sequence analysis, which no longer confines the assay to a small mutation set, has been evaluated and reported at 92–100% in individuals from many ethnic groups. Table 1 summarizes published detection rate data by enzyme, targeted mutation, and sequence analyses in the AJ and non-AJ population.

**Table 1.** Carrier and Disease Detection Rates for Tay-Sachs Disease

	*Biochemical*		*Targeted mutation analysis*		*NGS*	
*Publication*	*AJ (%)*	*Non-AJ (%)*	*AJ (%)*	*Non-AJ (%)*	*AJ (%)*	*Non-AJ (%)*
Kaback *et al.* (1993)	—	—	92–98	46	—	—
Bach *et al.* (2001)	93–99	—	99	—	—	—
Monaghan *et al.* (2008)	97–98	—	93–95	—	—	—
Schneider *et al.* (2009)	—	—	89	—	—	—
Park *et al.* (2010)	—	92	—	59	—	92
Strom *et al.* (2013)	—	87	—	—	—	94
Hoffman *et al.* (2013)^a^	89		68–72		85 (without VUS) 100 (with VUS)	

^a^ Some participants reported partial Jewish ancestry.

AJ, Ashkenazi Jewish; NGS, next-generation sequencing; VUS, variants of uncertain significance.

With increasing population diversity and clinical utilization of pan-ethnic screening, optimal screening protocols are needed for TSD. In this study, we compared TSD carrier screening by enzymatic analysis to sequence analysis in the non-AJ population at Columbia University Medical Center.

## Materials and Methods

### Participants and testing protocol

The Columbia University Medical Center Department of Obstetrics and Gynecology (New York, NY) began routine pan-ethnic screening for TSD in 2013 as part of a larger protocol of expanded pan-ethnic carrier screening. Testing was performed as fee for service, typically paid for by third party payers. Test uptake was voluntary and genetic counseling was made available to all individuals tested.

**FIG. 1.**
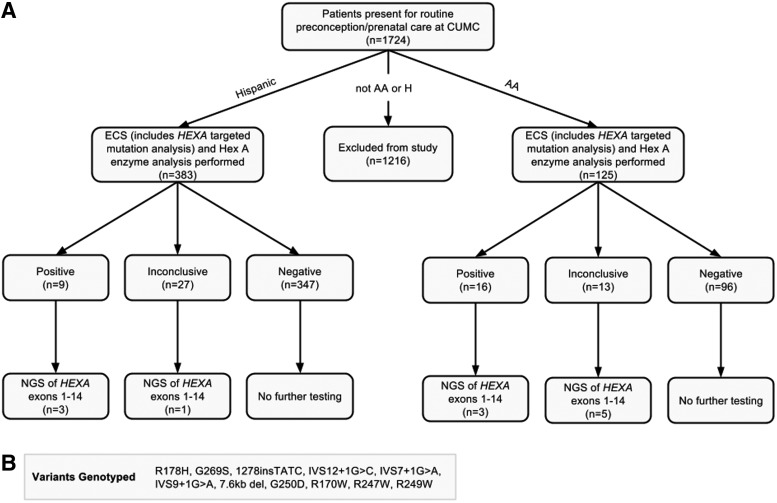
**(A)** Overall data set and analyses. **(B)** Nine mutations and two pseudodeficiency alleles genotyped as part of targeted mutation analysis. Five hundred eight patients of self-reported African and/or Hispanic descent are represented by 516 enzyme analyses. Six patients had an inconclusive enzyme analysis initially, which resolved to negative for three patients and positive for one patient. One patient had two inconclusive results, which resolved to positive after a third enzyme analysis. Three patients with negative repeat Hex A enzymatic results were not offered sequencing. AA, African/African American; CUMC, Columbia University Medical Center; ECS, expanded carrier screening; H, Hispanic; NGS, next-generation sequencing.

The patient population was diverse and to optimize TSD carrier detection, all individuals underwent screening simultaneously by targeted mutation analysis of nine pathogenic *HEXA* mutations and measurement of leukocyte Hex A activity levels (white blood cell [WBC] Hex A%). Paying particular attention to those patients that reported African, African American, and/or Hispanic ancestry (*n* = 508), for which carrier data scarcely exist, patients with a negative molecular analysis and a positive or inconclusive enzymatic result were offered sequencing of *HEXA* by the ordering provider, excepting three patients who had negative (noncarrier) enzymatic results upon repeat analysis (Fig. 1A). We retrospectively analyzed the results of these samples. All testing was performed as per the Department's clinical protocol, and results of the study were de-identified, providing exemption from institutional review board oversight. Results of molecular and enzymatic analyses for TSD carrier status in the population are reported.

### HEXA DNA analysis

*HEXA* mutation analysis of nine pathogenic mutations, including the 7.6 kb deletion common in French Canadians (De Braekeleer *et al.*, 1992), and the two pseudodeficiency alleles, R247W and R249W (Fig. 1B), was performed as part of the Family Prep Screen 1.0 at Counsyl (South San Francisco, CA). Screening for these mutations was done by high-throughput genotyping and has been previously described (Srinivasan *et al.*, 2010; Lazarin *et al.*, 2013).

Next-generation sequencing (NGS) of exons 1–14 in *HEXA* was performed as part of the Family Prep Screen 2.0, also at Counsyl. DNA from a patient's blood was isolated and then fragmented by sonication. The fragmented DNA was converted to an adapter-ligated sequencing library; samples are multiplexed and identified by molecular barcodes. Hybrid capture-based enrichment for *HEXA* targeted regions was performed on these multiplexed samples, after which NGS of the selected targets was performed with sequencing-by-synthesis on the Illumina HiSeq 2500 instrument (Illumina, San Diego, CA). Variants of uncertain significance (VUS) and benign variants were not reported to the patient as part of standard reporting protocol, but were examined for the purposes of this study. Variant classification at Counsyl is achieved through customized software analysis, which is consistent with guidelines from the American College of Medical Genetics and Genomics (ACMG; Richards *et al.*, 2008), and involves review of patient data (case reports and patient databases), population data, molecular functional data, mutational co-occurrence, protein structural analysis, conservation, *in silico* predictors, and internal data (Karimi *et al.*, 2015).

### Hex A enzyme analysis

Enzymatic analysis was mainly performed at Mount Sinai Genetic Testing Laboratory (New York, NY). Hex A activity and Hex A% activity were measured in leukocytes by a standard heat-inactivation fluorometric method using artificial 4-MU-*β*-*N*-acetyl glucosaminide substrate. The noncarrier range is 55.0–72.0% and the expected carrier range is <50% for leukocyte analysis. One enzyme assay (Sample 33) was performed at Mayo Medical Laboratory (Rochester, MN), where testing is done by a heat-inactivation fluorometric assay, and the noncarrier range is 63–75%.

## Results

Data were analyzed from 508 African/African American or Hispanic self-reported patients, who had screening between July 2013 and March 2014. Of these, enzyme analysis results were positive (“carriers”) for 25 (4.9%) patients, with a mean WBC Hex A% of 44.6%. Another 40 (7.9%) patients had inconclusive results with a mean WBC Hex A% of 52.8%, and 443 patients had negative results with a mean WBC Hex A% of 62.6%.

Of 73 total positive/inconclusive enzyme tests, five were repeat analyses after inconclusive results, leaving 68 unique patients. All 68 patients were negative by targeted mutation analysis (Fig. 1B), including negative results for the 7.6 kb deletion and pseudodeficiency alleles (R247W and R249W). Because three of the patients resolved as negative on further enzyme follow-up, 65 patients with non-negative enzyme results were eligible for sequence analysis, and 12 proceeded to sequencing (Fig. 1A). Of these 12, one patient positive by enzyme analysis had a likely deleterious mutation (NM_000520.4(HEXA):c.1510C>T), previously reported by Mules *et al.* (1992), and two patients had no detectable variants (Table 2). The remaining nine patients had between two and six known benign variants. No VUS were detected.

**Table 2.** *HEXA* DNA and Hex A Enzyme Result Summary

*Index no.*	*Ethnicity*	*Hex A result (WBC Hex A%)*	*NGS result (variant)*
Sample 6	African American	Inconclusive (50.9)	Known benign (c.1074-43C>G)
			Known benign (c.1074-94C>A)
			Known benign (c.1074-127A>T)
			Known benign (c.1306A>G)
			Known benign (c.1518A>G)
Sample 33^a^	African American	Positive (52.0)	Known benign (c.1074-94C>A)
			Known benign (c.1074-127A>T)
			Known benign (c.1306A>G)
			Known benign (c.1518A>G)
Sample 34	Dominican Republic	Positive (43.6)	Known benign (c.1306A>G)
			Known benign (c.1518A>G)
Samples 35 and 36	West Indian, Caribbean	Inconclusive (52.0 and 54.0)	Known benign (c.1074-43C>G)
			Known benign (c.1074-94C>A)
			Known benign (c.1074-127A>T)
			Known benign (c.1306A>G)
			Known benign (c.1518A>G)
Samples 43 and 44	African American, Puerto Rican, other Hispanic	Positive (52.4 and 45.9)	None
Sample 46	Hispanic	Inconclusive (52.1)	None
Sample 49	Hispanic	Positive (45.1)	Known benign (c.1306A>G)
			Known benign (c.1518A>G)
Sample 51^b^	African American	Inconclusive (51.0)	Known benign (c.1306A>G)
			Known benign (c.1518A>G)
Sample 55	Guyanese	Positive (36.4)	**Likely deleterious (c.1510C>T)**
			Known benign (c.1074-43C>G)
			Known benign (c.1074-94C>A)
			Known benign (c.1074-127A>T)
			Known benign (c.1195A>G)
			Known benign (c.1306A>G)
			Known benign (c.1518A>G)
Samples 56^b^ and 57	African American	Inconclusive (51.8 and 54.8)	Known benign (c.1306A>G)
			Known benign (c.1518A>G)
Sample 68	Senegalese	Positive (45.8)	Known benign (c.1074-43C>G)
			Known benign (c.1074-94C>A)
			Known benign (c.1074-127A>T)
			Known benign (c.1306A>G)
			Known benign (c.1518A>G)
Sample 73^b^	Nigerian	Inconclusive (51.5)	Known benign (c.1074-43C>G)
			Known benign (c.1074-94C>A)
			Known benign (c.1074-127A>T)
			Known benign (c.1195A>G)
			Known benign (c.1306A>G)
			Known benign (c.1518A>G)

Each row corresponds to one patient, and rows with multiple samples listed indicate that the patient had a second enzyme analysis performed due to the first being inconclusive. Allele names are with respect to NM_000520.4(HEXA). The bold value is the only abnormal DNA finding among our patient data.

^a^ Patient had Tay-Sachs enzyme analysis performed at Mayo Medical Laboratory, where the noncarrier range is 63–75%, and 52.0% is considered to be in the carrier range.

^b^ Patient also had Tay-Sachs enzyme analysis by plasma assay, which was in the noncarrier range (Samples 51, 56, and 73).

WBC, white blood cell.

## Discussion

Published data regarding mutations in the non-AJ population have been available for decades, and with the advent of NGS, data have been growing. Multiple studies (Tanaka *et al.*, 1990; Mules *et al.*, 1991, 1992; Levit *et al.*, 2010; Ribeiro *et al.*, 2011; Gort *et al.*, 2012) demonstrate that individuals of African and Hispanic ancestry tend to have pathogenic mutations other than the ones typically found in the AJ population. However, we did not identify any publications to date that have systematically evaluated the performance of the Hex A enzymatic assay in leukocytes, serum, or platelets in individuals of non-AJ descent. Hex A enzyme activity reference ranges have been established in the AJ population (Petersen *et al.*, 1983; Nakagawa *et al.*, 2012; Strom *et al.*, 2013), and these values have seemingly been applied to assess risk in all ethnic groups without further validation. In addition, recent data support the likelihood that average levels of Hex A activity are ∼5% lower in the African American population, which would explain higher rates of positive and inconclusive enzyme results (Neitzel *et al.*, 2015).

The rate of positive enzyme results in this set of African/African American and Hispanic patients (25/508) was significantly higher than the ∼1/300 TSD carrier frequency expected in the general low-risk population (Myrianthopoulos and Aronson, 1966) (*p* = 3.93 × 10^−21^, exact binomial test). *HEXA* sequencing was performed on six patients with positive enzyme results (of 25 total positive enzyme results), with only one patient carrying a deleterious variant; known deleterious mutations and VUS were not identified in any other patient, including the six patients with inconclusive enzyme results. Based on the observed carrier rate by NGS, if all positive enzyme results had been followed up by sequencing, we would expect to identify up to four patients with a deleterious variant. Up to four positive NGS results in the population of 508 individuals would be statistically consistent with the accepted 1/300 general low-risk population carrier frequency (*p* values of exact binomial test: 0.42, 1.0, 0.69, 0.24, 0.09, and 0.029 for 0, 1, 2, 3, 4, or 5 positive results, respectively). Therefore, our data set suggests that in the African/African American and Hispanic populations, the rate of positive results in the enzyme assay is inconsistent with the presumed (rare) carrier frequency of TSD, whereas the rate of NGS-positive results is consistent with current knowledge of the disease frequency.

We observed that the single patient with a deleterious variant had the lowest Hex A enzyme activity (Table 2). While our data set is too small to suggest ethnic-specific reference ranges, further investigation appears warranted.

Study limitations include the inability of the NGS assay to detect certain pathogenic variants (e.g., copy number variations other than the 7.6 kb deletion, inversions, or intronic variants). While these types of pathogenic variants may be present in the individuals with a positive/inconclusive enzyme analysis, to our knowledge, they have not been reported in affected individuals or are exceedingly rare. Another limitation is the sample size. As only 12/65 (18.5%) individuals proceeded to *HEXA* sequencing, it is possible that our results are not a generalizable sample of the population. However, it is of note that the positive rate by enzyme was significantly different from the expected rate, whereas the positive rate by NGS was consistent with population expectations. Thus, our experience further raises questions regarding the reliability of the enzyme assay in the non-AJ population.

## Conclusions

Our experience suggests, at minimum, ethnic-specific recalibration of Hex A enzyme reference ranges in the non-AJ population. Alternatively, NGS as the primary screening modality in the non-AJ population should be considered, as various publications have already demonstrated that detection rates by NGS are comparable to or better than those reported for enzymatic analysis. We continue to collect data characterizing non-negative enzyme results, which may reveal additional unrealized pathogenic variants or pseudodeficiency alleles. As our laboratory and others continue to classify variants and share data in public databases, clinical sensitivity of the NGS assay is expected to further increase, and NGS may be a realistic alternative screening protocol for certain populations in the near future.
